# Optical and Electrical Analysis of Annealing Temperature of High-Molecular Weight Hole Transport Layer for Quantum-dot Light-emitting Diodes

**DOI:** 10.1038/s41598-019-46858-6

**Published:** 2019-07-17

**Authors:** Young Joon Han, Kunsik An, Kyung Tae Kang, Byeong-Kwon Ju, Kwan Hyun Cho

**Affiliations:** 1 Micro/Nano Scale Manufacturing Group, Korea Institute of Industrial Technology (KITECH), 143, Hanggaul-ro, Sangnok-gu, Ansan-si 15588 Korea; 2 Department of Electrical and Electronics Engineering, College of Engineering, Korea University, 145, Anam-ro, Seongbuk-gu, Seoul 02841 Korea

**Keywords:** Electronic devices, Electrical and electronic engineering

## Abstract

In this study, we introduce optimization of the annealing conditions for improvement of hardness and hole transporting properties of high-molecular weight poly [9, 9-dioctylfluorene-co-N-(4-(3-methylpropyl)) diphenylamine] (TFB) film used as a Hole Transport Layer (HTL) of Quantum-dot Light-emitting Diodes (QLEDs). As annealing temperatures were increased from 120 °C to 150 °C or more, no dissolving or intermixing phenomena at the interface between HTL and Quantum-Dot Emission Layer (QDs EML) was observed. However, when the annealing temperatures was increased from 150 °C to 210 °C, the intensity of the absorbance peaks as determined by Fourier Transform Infrared (FT-IR) measurement was found to relatively decrease, and hole transporting properties were found to decrease in the measurement of current density - voltage (CD - V) and capacitance - voltage (C - V) characteristics of Hole Only Devices (HODs) due to thermal damage. At the annealing temperature of 150 °C, the QLEDs device was optimized with TFB films having good hardness and best hole transporting properties for solution processed QLEDs.

## Introduction

Colloidal Quantum-Dots (QDs), which are solution-processed nanoscale crystals used as semiconducting materials, have been extensively studied for their optical properties, which depend on the inherent size of the QDs^1–4^. QLEDs are attracting attention as a strong candidate for next-generation display industry technology, based on their advantages of high saturation, good stability, tunable luminescent color, and easy solution process^5–7^. However, there are technical problems that must be solved for the commercialization of QLEDs, including Highest Occupied Molecular Orbital (HOMO) level matching^8^ and the need to control the quenching of the QDs by ligands^9,10^. Above all, QDs have the limitation that they must be formed by solution process. With these limits, it is difficult to form multi-layered QLEDs well because the underlying layer can be partially dissolved by the organic solvent in the upper layer. The energy band balance between the HTL and the QD Emission Layer (QD EML) is important for controlling the hole tranport^11,12^, but the film properties may be deteriorated by dissolution or intermixing at the interface between two adjacent layers. These factors must be solved to improve QLED efficiency and life-time. Well-known methods to avoid dissolution and intermixing at the interface are the use of orthogonal solvents^13,14^ and a cross-linking^15^ approach for normal structured QLEDs and Organic Light-emitting Diodes (OLEDs). However, in the method using orthogonal solvents, the selection of materials is limited because the two adjacent layers should not use an organic solvent with each other. Also, the use of cross-linkable materials can limit the choice of materials that can be used for the best available hole transporting and injection properties of QLEDs. On the other hand, it has been reported that when a high-molecular weight organic material is used as an HTL in a solution process, dissolution does not occur even if EML is continuously formed using the organic solvent^16^. Only a simple annealing process is required after forming the film, without the need for complicated subsequent processes or additional materials. Recently, for the fabrication of solution processed QLEDs, Poly[N-vinylcarbazole] (PVK)^16^, TFB^17^ and Poly[triphenyldiamine] (poly-TPD)^18^, etc., were used as HTL, but there has been no accurate analysis of the annealing conditions of HTL in QLEDs. We carefully investigated the annealing conditions needed to achieve hardness without dissolving or intermixing, and good quality without damage to high-molecular weight HTL. We selected TFB as a high-molecular weight organic material (molecular weight, Mw = ~40,000) for use as an HTL. TFB is widely used as an HTL of OLEDs and QLEDs because it has good hole mobility^17,19^ and its HOMO level is located between those of Poly [3, 4‐ethylenedioxythiophene]: poly [styrene sulfonate] (PEDOT: PSS) and green QDs. In this study, the results of UV-vis and FT-IR analysis, which are commonly used for the analysis of organic materials, show that the molecular structure of TFB film was damaged at excessive annealing temperatures. In Optical Microscopy (OM) imaging performed with UV exposure and Focused Ion Beam Scanning Electron Microscope (FIB-SEM) analysis to observe sufficient annealing conditions to prevent dissolution of the TFB film, it has been shown that the thickness and quality of a TFB film with sufficient annealing temperature can be maintained without being dissolved by the QD EML. By analyzing the C - V characteristics of HODs, we have shown that the annealing temperature also affects the actual hole transporting properties; it has also been proved that the hole transport characteristics of the TFB film can be improved by annealing at a suitable temperature. The current - voltage - luminance (IVL) characteristics of QLEDs fabricated by spin-coating showed the same tendency as the previous optical and electrical analysis results.

## Results and Discussion

**Figure 1:**
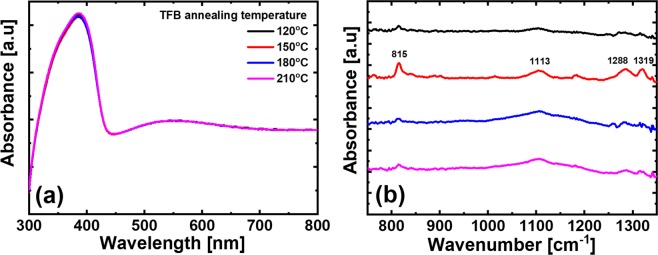
Optical analysis for TFB films (**a**) UV-vis absorption spectra and (**b**) FT-IR absorption spectra of TFB films of 120 °C, 150 °C, 180 °C, and 210 °C annealing conditions.

**Figure 2:**
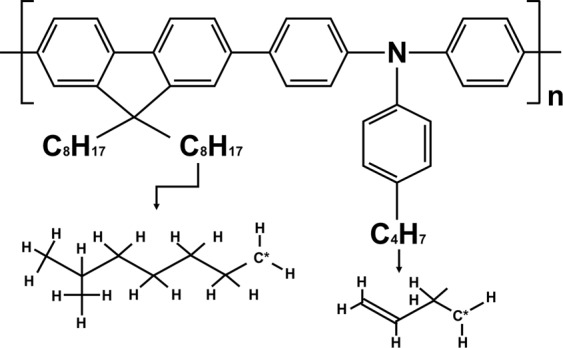
Molecular structure of TFB.

Figure 1 shows the optical analysis results for the TFB film according to the annealing temperature. In Fig. 1(a), the UV-vis analysis shows the absorbance of the TFB annealing temperature conditions. TFB films at each annealing temperature used for UV-vis analysis were spin-coated on glass substrates in the same conditions as those used for QLEDs fabrication; the thickness was about 30 nm. There is a peak at a wavelength of 390 nm, which is a commonly known TFB absorbance wavelength^20^. Based on these results, it was concluded that the annealing process of the TFB film had no significant effect on the composition and thickness of the TFB film^21^. Since the absorbance does not depend on the annealing temperature conditions, the concentration of TFB molecular crystals also was not changed. Figure 1(b) shows the absorbance spectra of TFB film, as analyzed by FT-IR. The same TFB film thickness and annealing conditions as the samples used in the UV-vis analysis were formed on the Si substrate. When an organic material is exposed to electromagnetic radiation, energy is absorbed at a specific wavelength and other wavelengths are passed through. This molecular vibration characteristic of infrared rays is useful for observing the molecular structure of an organic material and can be used to extract the absorption wavelength of a specific organic material. In this experiment, FT-IR analysis was used to observe the molecular structure of the TFB by annealing temperature change. As a result of FT-IR analysis, there was no prominent absorbance peak in the TFB film annealed at 120 °C, because the annealing temperature was not sufficiently hardening, and the evaporation of the organic solvent is not complete and the residual solvent is present. However, at the TFB film of 150 °C annealing condition, the intensity of absorbance peaks was increased at wavenumber of 815, 1113, 1288, and 1319 cm^−1^. To explain these absorbance peaks, the TFB molecular structure is shown in Fig. 2. In previously reported wavenumber peaks in organic materials irradiated by infrared in FT-IR analysis^22^, 815 peaks were found to be due to C-H bending out-of-plane vibrations of para-disubstituted benzene rings; 1113 peaks were due to the bending mode of C-C stretching between phenylene rings or the C-H bending mode of the side chains. The 1288 peak appears to be the C-C stretching mode between phenylene rings, finally, the 1319 peak is the vibration peak of the C-H bends. Absorbance peaks are summarized in Table 1. This shows that the TFB is stably hardened and thus maintains a robust molecular structure form of the 150 °C annealing condition. In the case of annealed TFB film at 180 °C and 210 °C, the intensity of absorbance peaks was relatively decreased compared to the 150 °C annealing condition. This is because the high annealing temperature has damaged the molecular structure of TFB.

**Table 1:** Main vibration peaks of TFB film extracted by FT-IR analysis.

Wavenumber [cm^−1^]	Explain for main vibration peaks
815	C-H out-of-plane vibration from 1,4-disubstituted benzene rings
1113	C-H bending mode of side chains
1288	C-C stretching modes between phenylene rings
1319	C-H bending

**Figure 3:**
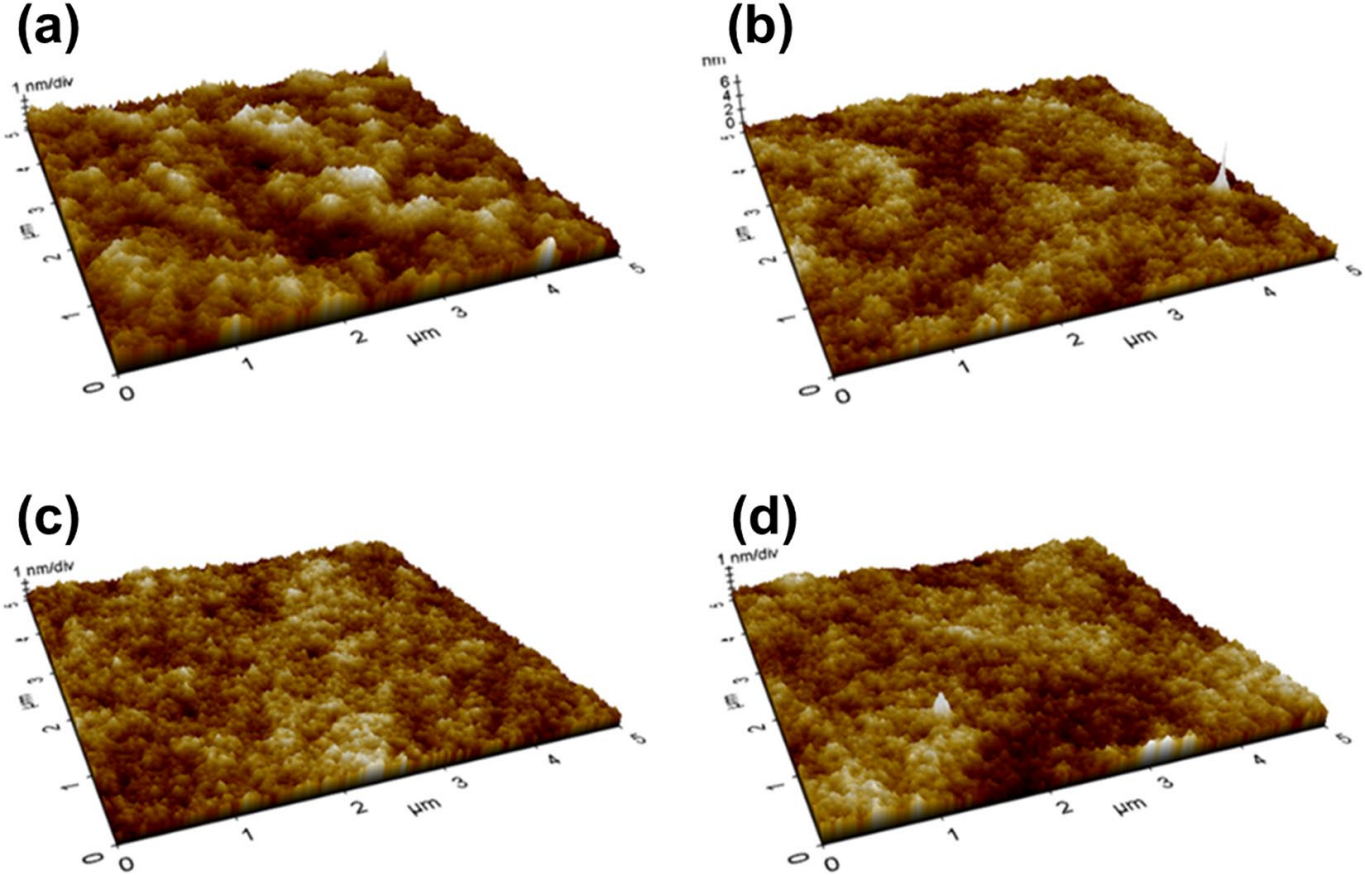
AFM images of TFB films of (**a**) 120 °C, (b) 150 °C, (**c**) 180 °C, and (**d**) 210 °C annealing condition; surface roughness RMS values for each annealing condition are 0.48 nm, 0.34 nm, 0.29 nm, and 0.36 nm, respectively.

**Figure 4:**
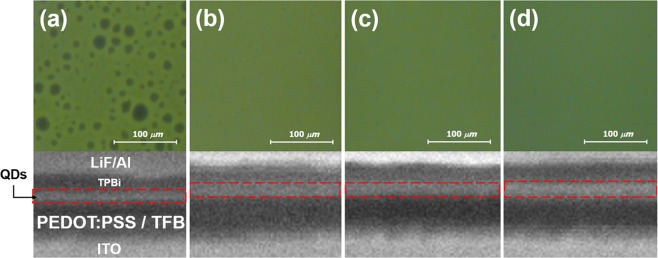
OM images with UV exposure of QDs film on the TFB film and the FIB-SEM images of the ITO/PEDOT: PSS/TFB/QD/TPBi/LiF/Al structures cross-section of (**a**) 120 °C, (**b**) 150 °C, (**c**) 180 °C, and (**d**) 210 °C annealing condition. The QDs film was annealed at 100 °C after spin-coating.

Figure 3 shows atomic force microscope (AFM) images of the surface of the TFB film for each annealing condition. The surface roughness root-mean-square (RMS) value of the TFB film of the 120 °C annealing condition was 0.48 nm, and the TFB film annealed at 150 °C, 180 °C and 210 °C were 0.34 nm, 0.29 nm and 0.36 nm, respectively. The TFB film with 120 °C annealing conditions have slightly higher surface roughness RMS values than TFB film with the other annealing conditions. It has been discussed in previous studies that an organic film with smooth and pinhole-free surface is obtained by increasing the annealing temperature of the spin-coated organic material^23^. As the annealing temperature and time are increased, the surface roughness RMS value of the organic film becomes gradually lowered, and the specific surface roughness RMS value is maintained depending on the type of the organic materials. In our experiment, there is no significant difference in the surface roughness RMS values of the TFB film of the 150 °C, 180 °C and 210 °C annealing conditions, and there is no significant difference in the properties of the QDs film formed on the TFB film. Figure 4 shows an OM image with UV exposure of the QD film formed on the TFB film; also shown is an FIB-SEM image of the cross-section of the ITO/PEDOT: PSS/TFB/QD/1, 3, 5-tris (2-N-phenylbenzimidazolyl) benzene (TPBi)/lithium fluoride (LiF)/Aluminum (Al) structure for each annealing condition. The QDs EML spin-coated on TFB films at all annealing conditions were annealed at 100 °C for 30 minutes. The boiling point of toluene, used to disperse the TFB, was reported to be about 110.6 °C. There will be relatively more residual toluene in the TFB film under the 120 °C annealing condition than in the other annealing temperature conditions. Thus, in Fig. 4(a), the dark spots on the surface of the QDs EML formed on the TFB film appear to be due to dewetting phenomena of the QDs film caused by interaction between the residual toluene of the TFB film and the hexane of the QDs solution during the spin-coating process of the QDs film. In detail, due to the residual toluene remaining locally in the TFB film, the surface energy of the TFB film is not uniform and appears to be localized dewetting on the TFB film surface in the QDs EML spin-coating process. Also, The FIB-SEM image in Fig. 4(a) confirms that the hardness of the TFB film under the 120 °C annealing condition is insufficient. The thicknesses of the PEDOT: PSS/TFB and QDs EML under the 120 °C annealing condition decreased by 9.1 nm and 9.6 nm, respectively, when compared with the 150 °C annealing condition. In Table 2, the thicknesses of the PEDOT: PSS/TFB and QDs EML depending on each TFB annealing condition are summarized. For the 120 °C annealing condition, the TFB film thickness reduction was due to dissolving phenomena originated from the hexane of the QDs solution during the spin-coating process, resulting in a paring of the TFB film and reduced thickness. The thickness reduction of the QDs EML appears to be due to intermixing between the TFB film and the QDs EML during both the spin-coating and annealing processes of the QDs EML. On the other hand, in the cases of the 150 °C, 180 °C, and 210 °C annealing conditions, dewetting was not found on surface of the QDs EML. In Table 2, it can be seen that the QDs EML or the TFB films under the annealing conditions of the 150 °C, 180 °C, and 210 °C are not different in thickness. This result shows that the TFB film annealed at 150 °C, 180 °C, and 210 °C were sufficiently hardened by annealing process, so that when the QDs EML was formed by spin-coating, almost no dissolution was caused by the organic solvent. In particular, the TFB film of the 150 °C annealing condition satisfy not only the hardening to prevent dissolution by the upper QDs EML, but also the robust molecular structures without damage, as shown in Fig. 1(b).

**Table 2:** Thickness of the PEDOT: PSS/TFB and QDs EML of the QLEDs structure using TFB film of each annealing condition.

Annealing temperature of the TFB film	120 °C	150 °C	180 °C	210 °C
QDs EML [nm]	25.6	34.7	34.3	35.8
PEDOT:PSS/TFB [nm]	66.2	75.8	74.6	74.9

**Figure 5:**
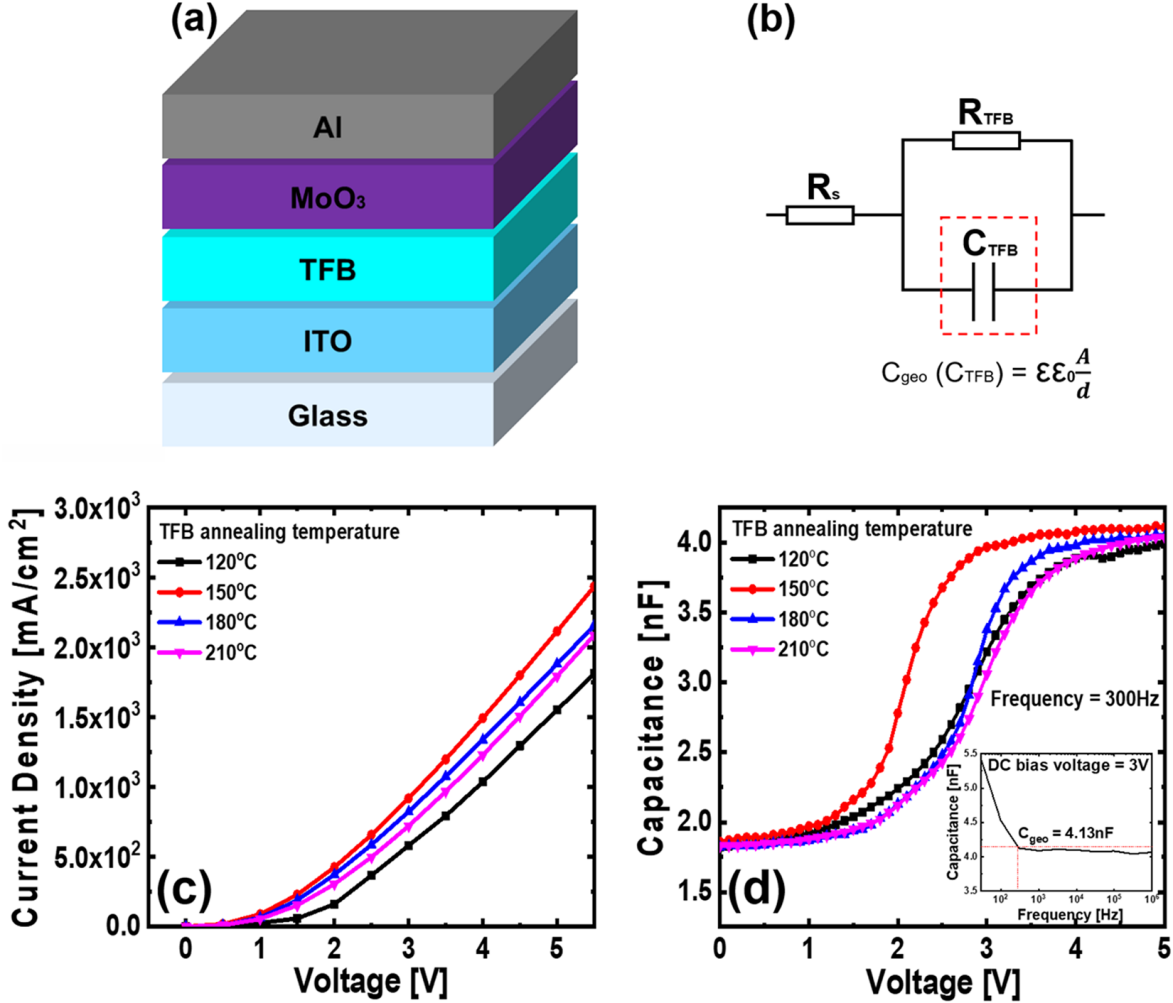
(**a**) Schematics and of the HODs structure. (**b**) Equivalent circuit, (**c**) CD - V characteristics, and (**d**) C - V characteristics (inset: C - f characteristics) of the HODs.

Previous capacitance analyses have also shown remarkable results in the electrical characterization of organic material devices. D. A. Ahn *et al*. have studied the effect of intermixing of organic HTL and EML interfaces on the performance of organic light-emitting diodes (OLEDs) by capacitance analysis^24^. We fabricated the HODs to investigate the hole transporting properties with the variation of annealing conditions of the TFB films. In Fig. 5(a), showing results of an analysis of HODs, to prevent electron injection from the Al cathode, molybdenum trioxide (MoO_3_) film was deposited on the TFB film by thermal evaporation before Al cathode deposition. In the equivalent circuit schematics of the HODs, shown in Fig. 5(b), R_s_ is the value obtained by integrating the resistance of the measuring equipment, the lead, and the electrode. R_TFB_ is the resistance in the TFB film; the capacitance value of the TFB film (C_TFB_) was calculated using the geometrical capacitance equation $({C}_{{\rm{geo}}}={\rm{\varepsilon }}\times {{\rm{\varepsilon }}}_{0}\times \tfrac{A}{d})$. The dielectric constant ε is 3.5, which is a valid value for organic materials^24,25^; the vacuum permittivity ε_0_ is 8.854 × 10^−12^ F/m; the active pixel size of the HODs *A* is 2 mm × 2 mm; and the thickness of the TFB film of HODs *d* is 30 nm. As a result, the capacitance of the HODs is calculated and found to be 4.13 nF. Figure 5(c) shows a comparison of the CD - V characteristics of the HODs for each annealing condition of the TFB films. As a result, when the annealing temperature of the TFB film increased from 120 °C to 150 °C, the CD - V curve shifted negatively. Although previous studies have reported a decrease in current density due to increased strength of the annealing process^24,26^, in this analysis, the current density increased as the annealing temperature of the TFB film increased from 120 °C to 150 °C. The reason for this phenomenon is that 120 °C is insufficient as the annealing temperature of the TFB film, and the residual solvent in the TFB film seems to have adversely affected the hole transporting properties. On the other hand, when the annealing temperature is increased from 150 °C to 180 °C and 210 °C, the CD - V characteristics curves shifted positively. This result can be matched with the FT-IR results in Fig. 1(b). It is expected that the hole transport characteristics of the TFB film deteriorated because high annealing temperatures of 180 °C and 210 °C damage the TFB molecules. Figure 5(d) compares the C - V characteristics of the HODs with the TFB film annealing temperature. The inset graph is a comparison of the capacitance - frequency (C - f) characteristics; we proceeded to find the frequency needed to reach the C_geo_ of the HODs and matched it at 300 Hz, so we analyzed the C - V characteristic at that frequency. In Fig. S1, in the low-frequency band (below 300 Hz), capacitance contribution by the shallow trap was observed under all annealing conditions. However, the difference in capacitance values at 30 Hz and 300 Hz was the smallest for the 150 °C annealing condition, and the stability of the capacitance value at the frequency band above 300 Hz was also excellent for the 150 °C annealing condition. In addition, the capacitance values of the HODs did not reach those of C_geo_ except for the 150 °C annealing condition. This result is attributed to the insufficient hardness of the TFB film during the fabrication of the HODs (at 120 °C annealing), or to deterioration of the TFB film quality due to destruction of the molecular structure (at 180 °C and 210 °C annealing). This result also demonstrates that the TFB film has the best properties with the 150 °C annealing condition. The C - V characteristics tend to be similar to the CD - V characteristics. This result shows that the 150 °C annealing conditions of the TFB films of the HODs are better than those of the other annealing conditions. The HODs with TFB film of 120 °C annealing condition had a capacitance value that increased from the earlier voltage compared to the HODs with TFB film of 180 °C annealing condition; however, the increase of the capacitance as the voltage increased was lower than that of the HODs with TFB film of 180 °C annealing condition. As a result, at the applied voltage of 3 V, the capacitance value of the TFB film at 180 °C annealing condition follows the capacitance value of the TFB film at 120 °C annealing condition. In addition, the HODs with TFB films of 120 °C and 210 °C annealing condition were not significantly different from the capacitance values. As a result of this experiment, the hole transporting efficiency of the TFB film increases when the annealing temperature increases from 120 °C to 150 °C and decreases when the annealing temperature increases from 150 °C to 180 °C or 210 °C. In addition, the effect of annealing temperature of the ITO electrode, depending on each annealing temperature, was analyzed by AFM analysis and sheet resistance measurement. As can be seen in Fig. S2 and Table S1, despite changing the annealing temperature of the ITO electrode, the sheet resistance and surface roughness RMS value were not significantly different.

**Figure 6:**
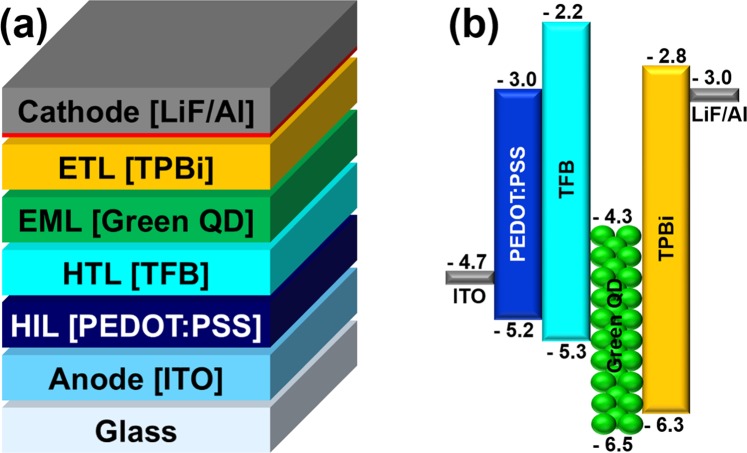
(**a**) Schematics of QLEDs structure, and (**b**) flat-band energy level diagram of each layer of the QLEDs.

**Figure 7:**
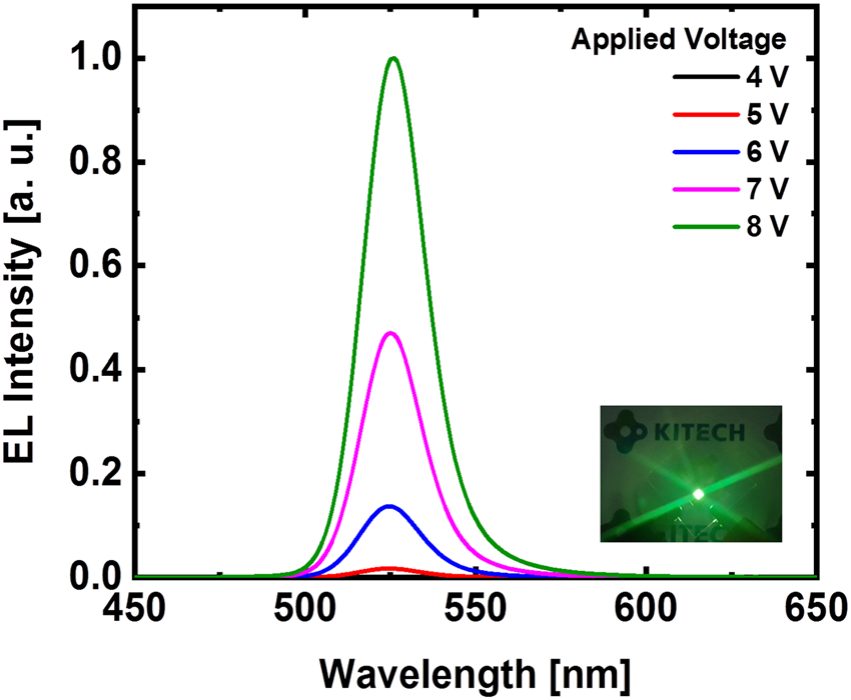
Evolution of EL spectra of QLEDs with increase of voltage from 4 V to 8 V. (inset: EL image under applied voltage of 5 V).

**Figure 8:**
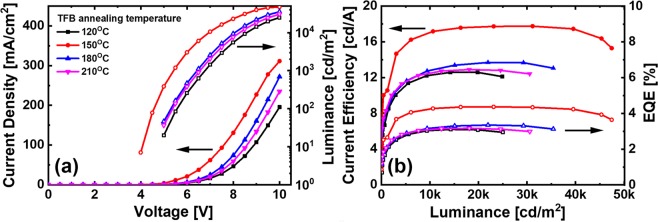
(**a**) Current density and luminance-voltage characteristics and (**b**) current efficiency and EQE-luminance characteristics of QLEDs.

Figure 6 shows the QLEDs structure and a flat-band energy level diagram of the materials used in QLEDs fabrication. The flat-band energy bend diagram in Fig. 6(b) shows that the TFB matches the HOMO levels of the PEDOT: PSS with QDs EML. TPBi is widely used as an ETL in the fabrication of OLEDs and QLEDs^27^ and has been studied as a means of improving the device performance by improving the interface contact as a buffer layer of the cathode electrode, preventing series resistance and current loss^28^. Therefore, although there is a loss in HOMO level matching of EML and the cathode electrode, the performance of the QLEDs is improved by using TPBi. In Fig. 7, electroluminescence (EL) spectra were measured by increasing the voltage applied to the fabricated QLEDs. The peak of the EL spectrum intensity is at the wavelength of 525 nm and the FWHM value is 21 nm. As the applied voltage increases, only the intensity increases: the peak wavelength of intensity remains unchanged and the FWHM value remains constant, so that stable EL emission of the QLEDs is measured. In the photograph of the EL emission of the inset, uniform EL emission is seen in the four light emitting portions of the QLEDs fabricated on the ITO coated glass substrate. The TFB film of the QLEDs used for the EL spectrum measurement was annealed at 150 °C. Figure 8 shows a comparison of the IVL characteristics of the QLEDs with different annealing temperature conditions for the TFB film. The IVL characteristics were measured in a normal atmosphere and at room temperature. In Fig. 8(a), the current density of the QLEDs of all annealing conditions was found to increase smoothly as the applied voltage increased, indicating that there is no leakage current affecting the IVL characteristics in any layers of the QLEDs structure. The current densities of QLEDs are quite consistent with the current densities of HODs with the function of the annealing temperature of the TFB film. The QLEDs with TFB film of 120 °C annealing condition was considered to be degraded not only by the lack of the hardening of TFB but also by the dewetting of the QDs EML. The QLEDs with the TFB film of 150 °C annealing condition had the highest current density because the hole transporting efficiency was increased by achieving the prevention of dissolution and intermixing phenomena by the optimized molecular structure of the TFB film. This is due to the fact that the current is well flowing because it has both achieved sufficiently hardening and a uniform QDs EML, which is also supported by the fact that the highest luminance was also achieved at QLEDs. In the case of the QLEDs with TFB annealed at 180 °C and 210 °C, the molecular structures of the TFB such as C-H bend or C-N bend were damaged due to the excessively high annealing temperature and the hole transporting efficiency of the TFB was lowered, resulting in a decrease in current density. This result is well suited to the results of the capacitance analysis in Fig. 5. The luminance was also highest at the annealing condition of 150 °C. Figure 8(b) shows the current efficiency and external quantum efficiency (EQE) versus the luminance, and the QLEDs with TFB film of 150 °C annealing condition have the best performance. The luminance, current efficiency, and EQE of the QLEDs with TFB annealed at 150 °C were 47,410 [cd/m^2^], 17.69[cd/A], and 4.36[%], respectively.

## Conclusions

In conclusion, we have used annealing temperature experiments to improve the hardness and dissolubility of high-molecular weight TFB films. Using UV-vis and FT-IR analyses, it was observed that the molecular structure of the TFB film of 150 °C annealing condition was not damaged and no dissolution was caused by the upper QD EML. Such a TFB film characteristic contributes to preventing degradation of electrical characteristics as an HTL, and thus it is possible to obtain an optimized hole transporting properties result. Finally, using solution-process HTL, we fabricated QLEDs using TFB film of optimized annealing condition; the IVL characteristics were measured to verify that the annealing temperature of the TFB film was an important factor in the performance of the QLEDs. Based on our experimental results, we conclude that excessive increase of annealing temperature of HTL has a non-negligible influence on the QLEDs characteristics. In addition, we expect to be able to study organic materials in more depth by combining optical and electrical analysis.

## Methods

### Materials

The indium-tin-oxide (ITO) coated glass substrate was sonicated for 1 hour in acetone and for 30 minutes in isopropyl alcohol (IPA). Next, UV-ozone treatment was performed for 20 minutes to make the surface energy of the ITO coated glass surface uniform, thereby improving the uniformity and characteristics of the film formed by the solution process. Poly [3,4‐ethylenedioxythiophene]: poly [styrene sulfonate] (PEDOT: PSS) (Clevios P VP AI 4083, Heraeus Co. Germany), used as hole injection layer (HIL), was mixed with IPA at a ratio of 1: 1 and mixture was sonicated for 5 minutes. By mixing IPA with PEDOT: PSS, the conductivity and hole injection efficiency of PEDOT: PSS were improved. poly [9,9-dioctylfluorene-co-N-(4-(3-methylpropyl)) diphenylamine] (TFB) (purchased from OSM Co., Korea) used as the HTL was dispersed in toluene at a concentration of 8 mg/ml and stirred at 50 °C and 400 rpm for 24 hours. The QDs had a CdZnSeS core/ZnS shell structure, and the surface ligand was formed by mixing trioctylphosphine and oleic acid (Purchased from In-visible Co., Korea). QDs dispersed in hexane at a concentration of 20 mg/ml was stirred at room temperature and 400 rpm for 2 hours before the spin-coating process. The other chemicals (toluene, hexane) purchased from Sigma-Aldrich Co. were used without further purification unless mentioned otherwise.

### Fabrication of HODs

The HODs was fabricated for capacitance characteristics analysis, the thicknesses of each layer are ITO (180 nm)/TFB (30 nm)/MoO_3_ (10 nm)/Al (100 nm). The TFB used dielectric layer for HODs was spin-coated at 4000 rpm for 30 seconds and annealed at each temperature (120 °C, 150 °C, 180 °C, and 210 °C) for 30 minutes on the cleaned ITO coated glass substrate. Then, MoO_3_ was deposited to 10 nm at 1 Å/sec and then Al was deposited to 2 Å/sec at 100 nm by thermal evaporation. The thermal evaporation process was conducted under vacuum atmosphere of 2 × 10^−7^ Torr.

### Fabrication of QLEDs

PEDOT:PSS was spin-coated at 2000 rpm for 30 seconds and then annealed at 100 °C for 30 minutes on the cleaned ITO coated glass substrate. TFB was spin-coated at 4000 rpm for 30 seconds and annealed at each temperature for 30 minutes. The QDs were spin-coated at 3000 rpm for 30 seconds and annealed at 100 °C for 30 minutes. The solution process was carried out in normal atmosphere and at room temperature. Next, 1,3,5-tris (2-N-phenylbenzimidazolyl) benzene (TPBi), used as the electron transport layer (ETL), lithium fluoride (LiF), and aluminum (Al) were sequentially deposited by thermal evaporation. TPBi was deposited to a thickness of 25 nm at 0.8 Å/sec. To match the lowest unoccupied molecular orbital (LUMO) level between the ETL and the cathode electrode^29^, a LiF layer was deposited to a thickness of 1 nm at 0.5 Å/sec. Finally, Al, used as a cathode electrode, was deposited at 2 Å/sec to a thickness of 100 nm. The thermal evaporation process was conducted under vacuum atmosphere of 2 × 10^−7^ Torr.

### Characterization and measurement

UV-1800 (SHIMADZU Co., Japan) was used to measure the UV-vis absorbance of TFB films, and Nicolet5700 (Thermo electron Co., USA) was used to measure the FT-IR absorbance peaks of TFB films. M6100 OLED I-V-L Test System (McScience Co., Korea) was used to measure the current - voltage - luminance (IVL) and current density - voltage (CD - V) characteristics of QLEDs and HODs, respectively. Agilent 4284a Precision LCR meter (Agilent technologies, USA) was used to measure the capacitance - frequency (C-f) and capacitance - voltage (C-V) characteristics of the HODs.

## Supplementary information

Supplementary Information
