# Supplementary material for: Optical and Electrical Analysis of Annealing Temperature of High-Molecular Weight Hole Transport Layer for Quantum-dot Light-emitting Diodes
Source: Sci Rep. 2019 Jul 17;9:10385. doi: 10.1038/s41598-019-46858-6 (PMC6637245; doi:10.1038/s41598-019-46858-6)
Supplement: Supplementary file 1 — Supplementary Information [file 41598_2019_46858_MOESM1_ESM.docx]

**Supplementary Information**

**Optical and Electrical Analysis of Annealing Temperature of High-Molecular Weight Hole Transport Layer for Quantum-dot Light-emitting Diodes**

# Young Joon Han^1, 2^, Kunsik An^1^, Kyung Tae Kang^1^, Byeong-Kwon Ju^2, *^, Kwan Hyun Cho^1, *^

^1^Micro/Nano Scale Manufacturing Group, Korea Institute of Industrial Technology (KITECH), 143, Hanggaul-ro, Sangnok-gu, Ansan-si, 15588, Korea

^2^Department of Electrical and Electronics Engineering, College of Engineering, Korea University, 145, Anam-ro, Seongbuk-gu, Seoul, 02841, Korea.

_________________________________________________________________________________

*Corresponding author: [bkju@korea.ac.kr](mailto:bkju@korea.ac.kr) (B.K. Ju), [khcho@kitech.re.kr](mailto:khcho@kitech.re.kr) (K.H. Cho)


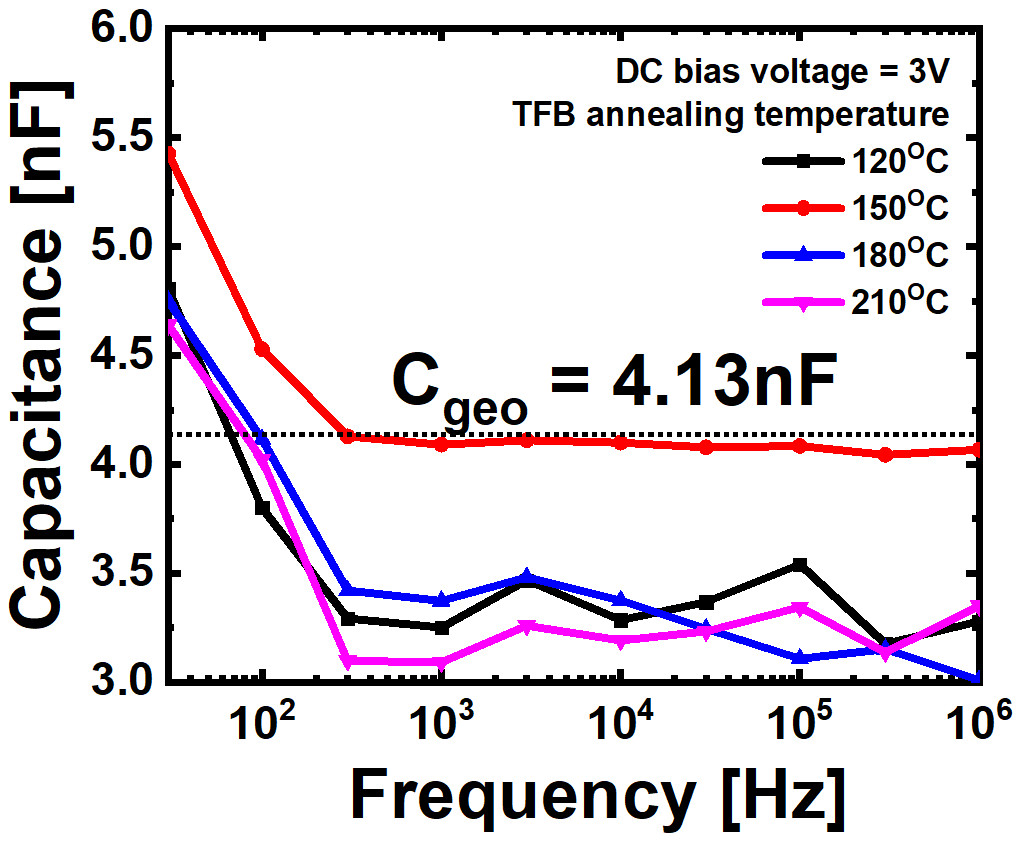


**Figure S1. Capacitance-frequency measurement of the HODs using the 120 °C, 150 °C, 180 °C, and 210 °C annealed TFB film.**


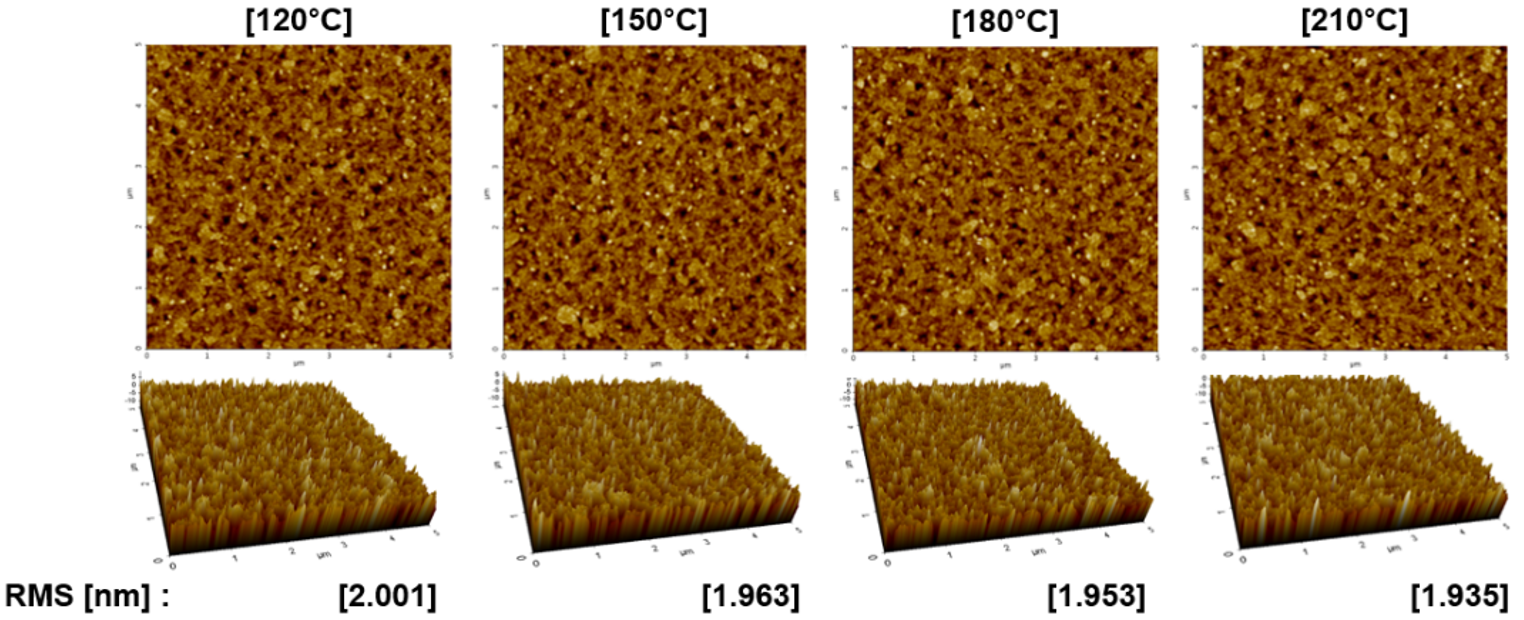


**Figure S2. AFM analysis of the ITO film with 120 °C, 150 °C, 180 °C, and 210 °C annealing temperature.**


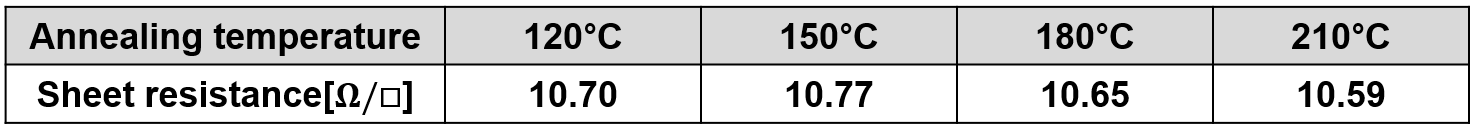


**Table S1. Sheet resistance measurement of the ITO film with 120 °C, 150 °C, 180 °C, and 210 °C annealing temperature.**
